# The Millimeter-Wave Radar SLAM Assisted by the RCS Feature of the Target and IMU

**DOI:** 10.3390/s20185421

**Published:** 2020-09-22

**Authors:** Yang Li, Yutong Liu, Yanping Wang, Yun Lin, Wenjie Shen

**Affiliations:** School of Information Science and Technology, North China University of Technology, Beijing 100144, China; liyang_ncut@ncut.edu.cn (Y.L.); LiuYutong_ncut@outlook.com (Y.L.); ylin@ncut.edu.cn (Y.L.); 15234183106@163.com (W.S.)

**Keywords:** millimeter-wave radar, simultaneous localization and mapping, radar cross section, density-based spatial clustering of applications with noise, correlative scan matching

## Abstract

Compared with the commonly used lidar and visual sensors, the millimeter-wave radar has all-day and all-weather performance advantages and more stable performance in the face of different scenarios. However, using the millimeter-wave radar as the Simultaneous Localization and Mapping (SLAM) sensor is also associated with other problems, such as small data volume, more outliers, and low precision, which reduce the accuracy of SLAM localization and mapping. This paper proposes a millimeter-wave radar SLAM assisted by the Radar Cross Section (RCS) feature of the target and Inertial Measurement Unit (IMU). Using IMU to combine continuous radar scanning point clouds into “Multi-scan,” the problem of small data volume is solved. The Density-based Spatial Clustering of Applications with Noise (DBSCAN) clustering algorithm is used to filter outliers from radar data. In the clustering, the RCS feature of the target is considered, and the Mahalanobis distance is used to measure the similarity of the radar data. At the same time, in order to alleviate the problem of the lower accuracy of SLAM positioning due to the low precision of millimeter-wave radar data, an improved Correlative Scan Matching (CSM) method is proposed in this paper, which matches the radar point cloud with the local submap of the global grid map. It is a “Scan to Map” point cloud matching method, which achieves the tight coupling of localization and mapping. In this paper, three groups of actual data are collected to verify the proposed method in part and in general. Based on the comparison of the experimental results, it is proved that the proposed millimeter-wave radar SLAM assisted by the RCS feature of the target and IMU has better accuracy and robustness in the face of different scenarios.

## 1. Introduction

In recent years, with the popularity of robots, drones, unmanned driving, and Virtual Reality/Augmented Reality (VR/AR), Simultaneous Localization and Mapping (SLAM) technology has also become also well-known and is considered to be one of the key technologies in these fields. SLAM technology was first proposed in the field of robots. It refers to robots which start from unknown locations in unknown environments, locate their positions and postures through repetitive observations of environmental features during their movements, and then construct an incremental map of the surrounding environment according to their own positions, so as to achieve the purpose of simultaneous localization and mapping. Due to the important academic value and application value of SLAM, it has always been considered as the key technology to achieve fully autonomous mobile robots.

At present, the mainstream SLAM sensors include two types: Lidar and optical camera. Lidar has high precision and can accurately model the three-dimensional environment, but it lacks the ability to cope with special weather conditions, such as rain, fog, smoke, etc. The advantage of an optical camera is that it has a wealth of information. The disadvantage is that it is severely disturbed by environmental and light conditions as well. However, the millimeter-wave radar has the ability to work all day and in all weather conditions [1]. In addition, the millimeter-wave radar can also gain information that lidar and cameras cannot, such as target speed and Radar Cross Section (RCS). RCS describes the intensity of the radar echo. It is a comparison of the scattered power density at the radar receiver with the incident power density at the target. This information can provide additional assistance for the achievement of SLAM. In addition, the price of the millimeter-wave radar is cheaper than that of lidar. Therefore, using the millimeter-wave radar as a SLAM sensor has obvious advantages and practical significance.

However, due to the data characteristics of the millimeter-wave radar, the application of SLAM also faces special challenges. First, the data volume of the millimeter-wave radar is very small, much lower than that of lidar. Second, the error of the millimeter-wave radar data fluctuates greatly. This is due to the characteristics of the radar system. In this paper, the data with large error fluctuation are called low-precision points. Third, the detection of millimeter-wave radar is easily distorted, because the multipath effect occurs frequently [2], especially in environments with many strong reflectors or in confined spaces. The distorted detection is called “outlier” in this paper.

The SLAM’s accuracy of localization and mapping are largely dependent on the data quality of the sensor, and the millimeter-wave radar data have a large number of low-precision points and outliers, which seriously affect the performance of SLAM. Therefore, in the data preprocessing of the millimeter-wave radar, it is very necessary to filter out low-precision points and outliers. For low-precision points, this paper uses the region of interest (ROI) for filtering. For outliers, a clustering algorithm can be used to filter out them. The clustering algorithm can identify the similarity between the individuals within the same class and the difference between the individuals in the cluster. In the clustering algorithm, selecting the appropriate threshold can separate the target point cloud from other clutter points. Common clustering algorithms include partition-based clustering, such as k-means algorithm [3]; hierarchical clustering, such as BIRCH [4]; clustering based on probability distribution, of which the typical representative is the EM algorithm [5]; and density-based clustering, of which the typical representative is the DBSCAN [6]. The density-based clustering algorithm defines the class as the largest set of points connected by density. It does not classify according to distance, and it overcomes the limitation that only “circular” clusters can be found based on the distance discrimination criterion. It can find the class with any shape or size. Compared with other clustering algorithms, DBSCAN not only does not need to input the number of clusters in advance, but also can automatically identify outliers and put them into a single class. Based on the above advantages, this paper uses DBSCAN to cluster millimeter-wave radar data, and then selects the target point cloud body in the clustering result to achieve the filtering of outliers. When using DBSCAN to cluster millimeter-wave radar data, usually only the two-dimensional Cartesian position features of the target are used, such as those described by the authors of [7,8], but due to the unevenness of the millimeter-wave radar data density, relying on the location features sometimes cannot accurately identify outliers. The authors of [9] used the speed characteristics of the target, but in a static environment, the target speed was 0, and only the position characteristics were actually used. In addition, DBSCAN has two parameters that need to be determined artificially: *e* and *MinPts* [6]. For two-dimensional data, many methods of adaptively determining parameters have been proposed, such as those described by the authors of [10,11,12,13]. However, for multidimensional data, there are few methods for adaptively determining parameters. JORG SANDER et al. [14] proposed a *MinPts* adaptive determination method for data of any dimension, but the parameter *e* still needs to be determined manually. In order to solve the above problems, this paper introduces the RCS feature of the target when using DBSCAN to improve the accuracy of identifying outliers and achieve the adaptive determination of parameters.

In the earliest applications of millimeter-wave radars for SLAM, high-visibility radar reflectors with known positions were processed by extended Kalman filtering (EKF) [15]. The reflector was later used as a landmark in SLAM based on EKF [16]. The main disadvantage of landmark-based SLAM is that it depends on extracting appropriate landmarks, which is difficult for millimeter-wave radars with low-range, angular resolution and a large amount of noise. A SLAM method based on scan matching used the Fourier-Mellin transform to match successive radar scans, but the radar signal was interpreted as a 360° power spectrum [17]. In the latest research, some millimeter-wave radar SLAM methods based on neural networks [18,19,20,21] have been very popular. By embedding a differentiable point-based motion estimator inside the architecture, the authors of [22] learned key point locations, scores, and descriptors from localization error alone, while the authors of [23] provided the same level of interpretability as established scan-matching methods and allowed for a principled derivation of uncertainty estimates. The authors of [24] proposed RadarSLAM, which was composed of pose tracking, local mapping, loop closure detection, and pose graph optimization, enhanced by novel feature matching and probabilistic point cloud generation on radar images. Almost all of these papers used neural networks to extract image features from radar image and require data for training. The authors of [25] proposed a new point association technique to match the sparse measurements of the low-cost millimeter-wave radar. It also used Recurrent Neural Network (RNN) to estimate the pose. The SLAM method based on cellular radio infrastructure is a very interesting direction, since cellular signals are ubiquitous, inexpensive to obtain and process, and require no additional hardware. The reason why cellular positioning has never been considered lies in its poor accuracy. As the fifth generation (5G) millimeter-wave wireless communication system matures, 5G SLAM overcomes this limitation [26,27]. The authors of [27] used message passing (MP) and the Kuhlman Kalman filter (CKF), which preserved the accuracy while greatly reducing the complexity. The authors of [28] put forth the idea of placing massive antenna arrays in smartphones or tablets, thus realizing a high-definition and low-cost personal mobile radar. Based on the idea of personal radar, which aimed to solve the problem that the antennas were far from having a pencil beam, the authors of [29] proposed an ad-hoc occupancy grid method for mapping, where the array radiation characteristics were accounted for in the observation model. The authors of [30] introduced a channel model for personal radar applications. It aimed at characterizing the channel from both a temporal and an angular perspective by exploiting a 2D CLEAN-like technique to extrapolate the multipath components and a K-means algorithm for clustering. These methods are similar to the millimeter-wave radar SLAM because they are all based on millimeter-wave technology.

Many lidar-based SLAMs abandon the problem of landmark extraction by directly matching point clouds [31,32,33]. Although the point cloud matching method has been successfully used in radar self-motion estimation [34] and location [35], it is rarely used in SLAM based on the millimeter-wave radar. Iterative closest point (ICP [36]) is widely used in lidar point cloud matching, and the standard ICP algorithm only considers “point-to-point” geometric information when iteratively searching for the closest point. However, in challenging environments (such as dynamic environments and highways), ICP cannot achieve accurate point cloud matching. Nowadays, improved ICP algorithms have been proposed, such as iterative closest point (PLICP) for “point-to-line” matching [37]. It can converge in a limited number of steps at a quadratic rate. The authors of [38] used the maximum iteration closest point (Go-ICP) to accelerate conventional ICP. However, this type of ICP and its improved methods are imperfect because they are sensitive to local minima. Poor initial estimates can lead to erroneous data associations and differences [39]. One method to find the local optimal value on the linear interpolation graph using the Gauss–Newton method was proposed by the authors of [40]. Normalized distribution transformation (NDT) [41] is a typical algorithm commonly used for 2D lidar scan matching. Gauss–Newton and NDT are effective local scan matching algorithms, which rely on accurate initial guess, but if the initial guess is far from the ground truth, they are also prone to fall into the local optimal state. Correlative Scan Matching (CSM) [39] is a global scan matching algorithm, which avoids the problem of local extremums, because it does not believe in the global maximum value found by local search and searches a reasonable entire area, which is derived from a priori, or can be obtained from other sensors such as motion commands or Odometry. Various methods [42,43] have been proposed to improve CSM’s lidar scan matching for 2D performance. However, when using CSM to process millimeter-wave radar data, a good position estimate cannot be obtained. This is because CSM only matches two adjacent scan point clouds, which is a “Scan to Scan” point cloud matching method. Since the data volume of the millimeter-wave radar is too small, and the accuracy of the data is also low, CSM cannot obtain accurate estimation results, and the cumulative error also increases. In order to solve the above problems, this paper proposes an improved CSM method which changes CSM to the “scan to map” point cloud matching method, and the RCS of the targets was introduced to improve the accuracy of point cloud matching.

**Figure 1:**
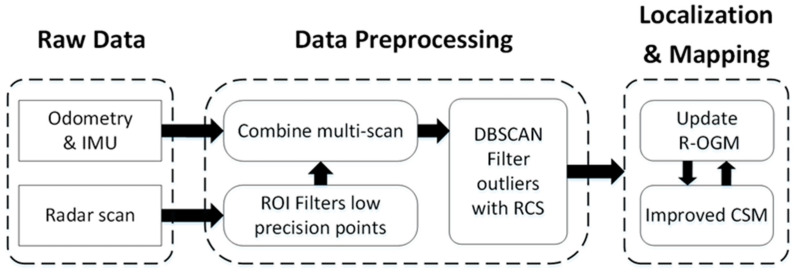
Composition of millimeter-wave radar Simultaneous Localization and Mapping (SLAM) assisted by the Radar Cross Section (RCS) features of the target and Inertial measurement unit (IMU).

In this paper, a kind of millimeter-wave radar SLAM assisted by the RCS feature of the target and IMU is proposed. It main contributions consist of two points. First, we use DBSCAN with the RCS feature of target to filter out outliers. Second, an improved CSM method is proposed to improve the accuracy of point cloud matching. The detailed composition of the system is shown in Figure 1. In Section 2, the preprocessing method of millimeter-wave radar data is introduced in detail. First, the error distribution of the target position measurement within the radar detection range was analyzed to obtain the ROI of high precision points, thereby filtering out low precision points. Then, using the Odometry and Inertial measurement unit (IMU), the combined multiframe scanning data of the radar was transformed to “Multi-scan”. This not only increased the number and density of point clouds for subsequent point cloud matching, but also enhanced the robustness of SLAM in a degraded environment. Finally, we used DBSACN to filter outliers of radar data. In this paper, the vectors of multidimensional features of millimeter-wave radar data are constructed, including the Cartesian localization feature and RCS features of the target. Here, we used the Mahalanobis distance to measure the similarity between the vectors, and achieved the self-adaptive determination of algorithm parameters. In Section 3, this paper proposes an improved CSM algorithm for the data characteristics of millimeter-wave radar to improve the accuracy and robustness of the radar point cloud matching algorithm. This is a “Scan to Map” method, which matches the radar point cloud with the local submap of the global grid map and achieves the tight coupling of localization and mapping. In addition, when constructing the global grid map, the RCS target was combined, which is called “RCS-Occupancy Grid Map (R-OGM)” in this paper. Three improved CSM methods were proposed, and the effectiveness of the third method is verified In Section 4. In Section 4, three groups of actual data are used to analyze and verify each link and the overall system. The comparison of the experimental results proves that the millimeter-wave radar SLAM proposed in this paper had good accuracy and robustness.

## 2. Radar Data Preprocessing

In order to better estimate the radar pose and map, high-quality radar data are necessary, especially when compared with lidar. High-quality radar data contain two conditions: High precision of position and nonoutlier. In the process of data preprocessing, it is necessary to select high-quality data for accurate point cloud matching. This ultimately determines the accuracy of SLAM positioning and composition. From the perspective of long-term operation, data preprocessing also reduces the burden of the computer, because it reduces the amount of storage and computing on the point cloud. In this section, first, the error distribution of target position measurement is analyzed, and ROI with simple shape is selected to filter low-precision points. Then, the continuous radar scanning are combined into “Multi-scan” by Odometry and IMU. Finally, the DBSCAN clustering algorithm is used to identify and filter outliers from the data in the ROI, and the parameters of the algorithm are determined adaptively. In clustering, this paper used the multidimensional features of the radar target, including the Cartesian position feature and RCS feature of the target, to improve the accuracy of outlier recognition. After the above steps, high-quality radar point cloud data were finally obtained.

### 2.1. Filter Low-Precision Points and Construct “Multi-Scan”

In recent years, vehicle-mounted millimeter-wave radars have become more and more popular due to their low cost and good robustness [44]. They usually give the measurement information of the detected targets directly. The original radar echo data contains targets in the form of point cloud, which is helpful for later processing. The vehicle-mounted millimeter-wave radar used in this paper can measure the position, speed, and RCS of the target. The radar detects *n* targets in each frame, and the measured value *Z_m_* of the *m-th* target contains multidimensional features, as follows:

(1) $$z_{m}=\left[r_{m}^{x},r_{m}^{y},v_{m}^{x},v_{m}^{y},rcs_{m}\right],1\leq m\leq n$$

**Figure 2:**
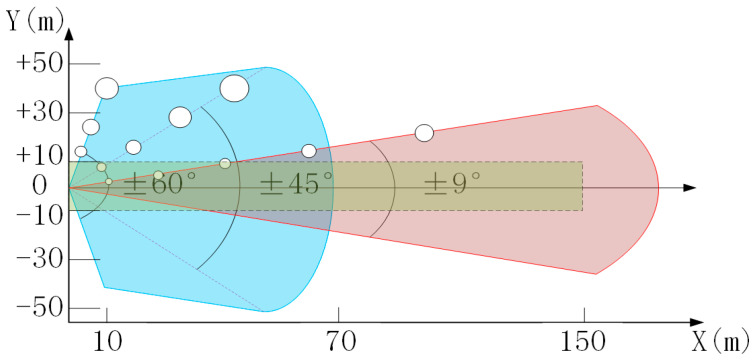
Distribution of target position measurement error in different radar detection ranges. The blue area represents the short-distance detection range, the red area represents the long-distance detection range, and the white circle represents the position measurement error of the target here. The larger the radius, the greater the error. The green area is the selected ROI. The target within the ROI has high position precision.

The measurements *r_m_^x^* and *r_m_^y^* of the target position of the millimeter-wave radar are the coordinates of the target relative to the radar in the Cartesian coordinate system. In different detection ranges of the radar, the measurement precision of target position is different. This precision is determined by the angle measurement accuracy and range accuracy of radar, and they are coupled with each other. Taking the millimeter-wave radar used in this paper as an example, the precision in different detection ranges is shown in Table 1. However, the visualization effect of Table 1 is too poor to be observed. Instead, the position measurement error distribution diagram within the radar detection range is drawn, as shown in Figure 2.

It can be clearly seen from the above figure that the position measurement errors of radar have different sizes at different angles and different distances. At the edge of the detection range, that is, the larger the angle and the farther the target is, the larger the position measurement error is, and the lower the position accuracy. In order to improve the accuracy of SLAM location and composition of the millimeter-wave radar, ROI was used to screen out the low-precision points in each frame of radar point cloud, and only the point clouds with high position precision in ROI were retained.

The amount of point cloud data that the single-frame millimeter-wave radar contains is very small, and they are sparse, which makes it difficult to match point clouds and remove outliers. Compared with the high-precision lidar (a single-frame scan contains thousands of points and the error is much smaller), the single-frame scanning point cloud of millimeter-wave radar usually contains only dozens of targets after filtering out the low-precision points. There are also some moving targets and outliers among them. Moving targets also reduce the accuracy of SLAM. Therefore, direct matching of continuous radar scan point clouds leads to poor results. On the contrary, this paper used Odometry and IMU data to combine *N* frames radar scanning point clouds as “Multi-scan”. There are three advantages of that method. First, using Odometry and IMU data, the absolute speed of the radar can be calculated, which is conducive to screening out moving targets. Second, “Multi-scan” improves the problem of small amount of point cloud data, which enables more accurate matching of point clouds. Third, “Multi-scan” also improves the sparsity of the point cloud and increases the density of the point cloud, so that the density-based DBSCAN can better filter out outliers.

In order to combine continuous *N* frames of scanning point clouds to form a “Multi-scan” and filter out moving targets, it is necessary to estimate the pose and velocity of the radar during continuous scanning. This paper used the extended Kalman filter (EKF) to fuse Odometry and IMU data. The filtered pose sequence [*O_t_*, *O_t+_*_1_, …, *O_t+N_*] was taken as the pose during continuous scanning, and they were used to combine *N* frames scanning. Since EKF outputted the absolute velocity of the radar and the radar also measured the relative velocity of the target, the absolute velocity of the target can be obtained. By setting the threshold, the irrelevant moving targets can be filtered out.

**Figure 3:**
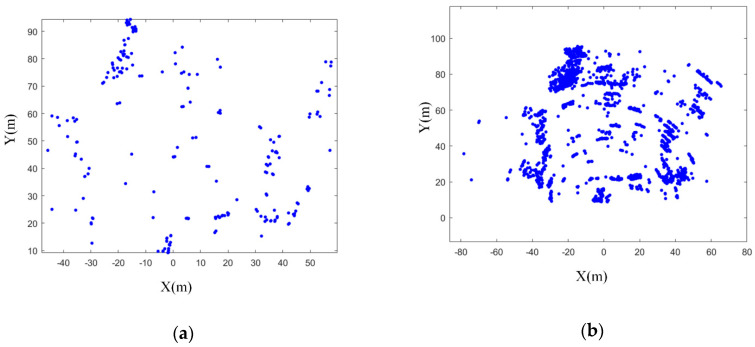
Point cloud of radar. (**a**) Single frame scanning point cloud of radar; (**b**) “Multi-scan” with *N* = 20.

This paper used Odometry and IMU to combine continuous scanning to form a “Multi-scan” with *N* = 20, as shown in Figure 3. In Section 2.2, DBSCAN is used to cluster the “Multi-scan” with *N* = 20 to identify and filter out outliers.

### 2.2. Outlier Removal by DBSCAN Based on RCS Feature

**Figure 4:**
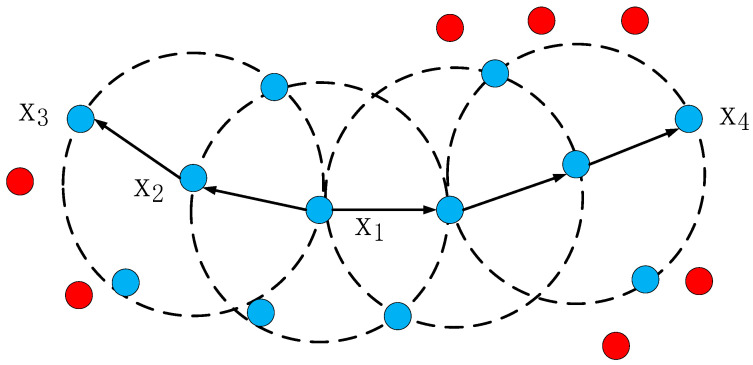
The above figure visually shows these concepts in DBSCAN: When *MinPts* = 3, the dotted circle is the e neighborhood, *X*_1_ is the core object, *X*_2_ is directly connected by *X*_1_ density, *X*_3_ is reachable by *X*_1_ density, and *X*_3_ is connected with *X*_4_ density.

DBSCAN is a famous density clustering algorithm, which uses a set of parameters about “neighborhood” to describe the compactness of sample distribution [6]. The parameters include neighborhood distance *e* and minimum number of neighborhood samples *MinPts*. The algorithm includes several concepts: Neighborhood, core object, density direct, density reachable, and density connected. The details are shown in Figure 4.

When DBSCAN is used to filter outliers from radar data, the two-dimensional position features *r_m_^x^* and *r_m_^y^* of targets are usually used, which are the position of targets observed by radar, as described by the authors of [7,8]. When the DBSCAN processing radar uses data with two-dimensional position features, it essentially divides point clouds according to Euclidean distance between targets. However, due to the nonuniformity of point cloud density in the millimeter-wave radar, outliers cannot be recognized and filtered out using only two-dimensional position features. Therefore, in this paper, the RCS feature of the target was added to cluster the “Multi-scan” after filtering out low-precision points, so as to filter out outliers.

From the data level, this paper considers that the nonoutlier point cloud should have similar features, that is, similar position, velocity, and RCS. The point that does not meet this condition is the outlier. DBSCAN usually uses Euclidean distance to measure the similarity, but this method is obviously not suitable for data with features higher than two dimensions. Because different features have different units and scales, and there may be correlation between features, it is obvious that Euclidean distance cannot achieve good results. Considering the use of other distances for similarity measurement, standardized Euclidean distance is a good choice. It makes the mean and variance of each dimension of data equal, but it does not consider the correlation between features. The characteristic of Mahalanobis distance is that it has nothing to do with the dimension and eliminates the interference of correlation between variables. It actually uses Cholesky transformation to eliminate the correlation between different features and the nature of different scales. Therefore, this paper chose Mahalanobis distance to measure the similarity between data.

The Mahalanobis distance is a distance based on sample distribution [45]. The physical meaning is the Euclidean distance in the normalized principal component space. Definition: There are *n* sample vectors *X*_1_*~X_n_*, the covariance matrix is denoted as *S*, and the mean is denoted as the vector *μ*. The Mahalanobis distance between the sample vector *X* and *μ* is expressed as:

(2) $$D\left(X\right)=\sqrt{{\left(X-\mu \right)}^{T}S^{-1}\left(X-\mu \right)}$$

The Mahalanobis distance between the vectors *X_i_* and *X_j_* is defined as:

(3) $$D\left(X_{i},X_{j}\right)=\sqrt{{\left(X_{i}-X_{j}\right)}^{T}S^{-1}\left(X_{i}-X_{j}\right)}$$

It should be noted that the covariance matrix *S* needs to be calculated, but in a static environment, the velocity feature of the target may be zero, which leads to the failure to calculate the covariance matrix. Since most of the moving targets had been preliminarily screened out in Section 2.1, the velocity feature was no longer considered as the input of clustering, and the position feature and RCS feature were used.

To input “Multi-scan” into DBSCAN, two parameters, *e* and *MinPts*, need to be input at the same time. The traditional DBSCAN needs to determine the parameters of *e* and *MinPts* artificially, but obviously this is not realistic for SLAM, so it needs to make a self-adaptive parameter determination method.

(4) $$X_{m}=\left[r_{m}^{x},r_{m}^{y},rcs_{m}\right],1\leq m\leq n$$

**Figure 5:**
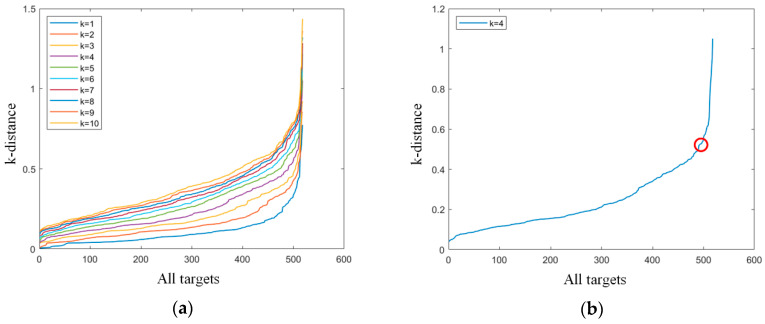
(**a**) Distance diagram with different *K* values; (**b**) Distance diagram with *K* = 4, where the circle is the position of “elbow”.

**Figure 6:**
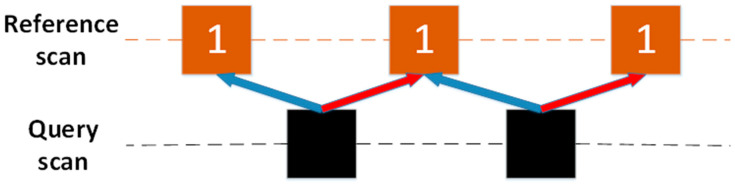
The original CSM matching diagram. In the reference scan, the number of blocks represents the occupation probability of the target. The original CSM will obtain two kinds of matching relations. The blue arrow indicates the first matching relationship, and the red arrow indicates the second matching relationship.

First, the three-dimensional vector *X_m_* of each target was constructed, such as in Formula (4), and the Mahalanobis distance between vectors was calculated. Then, the Mahalanobis distance between each target and all other targets was counted, and the Mahalanobis distance between each target and the k-nearest target was counted, respectively, which is called the “k-distance” in this paper. We recorded the k-distance of all targets, and drew the k-distance diagram in descending order. In this paper, the k-distance diagram with different *K* values were drawn, as shown in Figure 5a. At this time, we observed the k-distance figure, and a good value of *e* was the position of the “elbow” shown in the figure [6], as shown in Figure 5b. Here, *K* = 4 was taken as an example.

However, it is very difficult for the machine to identify the position of this “elbow,” which requires artificial selection. In this paper, we used the method described by the authors of [13] for reference. Since the k-distance between all vectors obeys Poisson distribution on the number axis, we used the maximum likelihood estimation method to estimate the parameters of the Poisson distribution of the whole k-distance, and the expected value is the value of *e*, as shown in Formula (5), where d represents the k-distance.

(5) $$e=\lambda =\bar{d}=\frac{1}{n}\sum_{i=1}^{n}d_{i}$$

At this time, a parameter selection problem was solved, that is, the automatic determination of *e* value was realized. However, in order to determine the value of *e*, a new parameter *K* was introduced. *K* is actually related to *MinPts*, such as in Formula (6). According to experience [14], when using multidimensional data, Formula (7) can be used to determine *MinPts*. Dimension is the feature number of the data.

(6) K=MinPts−1

(7) MinPts=2∗Dimension

At this time, the parameters of DBSCAN had been determined adaptively, the outlier identification and filtering had been realized, and the data preprocessing of the millimeter-wave radar had been completed. The verification experiment is shown in Section 4.

## 3. Tight Coupling of Localization and Mapping

### 3.1. Original CSM Method

The original CSM is a method to estimate radar pose by point cloud matching, which was proposed by Edwin B. Olson and applied to lidar [39]. When a new frame scan arrives (called query scan *Q* by the author), the rasterized lookup table is calculated in advance according to its previous frame scan (reference scan *F*). The essence of the rasterized lookup table is to use Gaussian kernel to blur *F*, which can make the map smoother for more accurate matching. F* is the grid near *F*, *Dist* is the Euclidean distance from *F* to F*, and then the occupancy probability of F* is calculated using Gaussian kernel, as shown in Formula (8), where *μ* is the expectation and *σ* is the variance.

(8) $$P(F^{*})=\frac{1}{\sigma \cdot \sqrt{2\pi }}e^{-\frac{{\left(Dist-\mu \right)}^{2}}{2\sigma^{2}}}$$

We need to estimate the radar pose *T_t_* corresponding to time *t*, which is a three-dimensional point including Δ*x*, Δ*y,* and Δ*θ*. Edwin B. Olson gave three estimation methods based on CPU.

A violent matching method is composed of three nested loops. *Q* is projected onto the rasterized lookup table. The grid score in each click is recorded, that is, the probability value of the corresponding grid. All scores are recorded, and the total score is obtained. The pose with the highest total score is considered to be the best estimation of pose, because the higher the total score, the better the matching degree. However, this method is slow.

The second method is to calculate 2D projection slices. By iterating Δ*θ* in the outermost loop, a lot of calculation time can be saved, and the query points will be rotated correctly. The two inner loops (for Δ*X* and Δ*y*) only translate the query points. This method is much faster than the violent method.

The third method is the multiresolution map method. The author constructed a map with two resolutions. The strategy of this method is to use low-resolution maps to quickly identify areas that contain global maximums and areas that do not contain global maximums. The purpose is to minimize the amount of search for high-resolution map. Then, the high-resolution map is used to search the area containing the maximum value. This multiresolution method is very fast, which makes real-time scanning matching possible.

### 3.2. Improved CSM Method

The original CSM is a “scan to scan” point cloud matching method, which only matches *Q* and *F* of two adjacent frames. However, for the following two reasons, the original CSM is not suitable for the millimeter-wave radar. The first reason is that the data volume of millimeter-wave radar is very small and sparse. Even if the “Multi-scan” has been constructed in the preprocessing process, it still cannot estimate the radar pose well when matching. The second reason is that the original CSM method gives the same occupancy probability to each point in the reference scan *F* when constructing the rasterization lookup table. For the millimeter-wave radar with low data precision, it leads to poor results of point cloud matching. As shown in Figure 6, the orange square represents the reference scan *F* and the black square represents the query scan *Q*. The numbers in the orange square are the occupancy probabilities assigned to the point cloud by the Gaussian distribution, and they are all equal to 1. Using the original CSM, we obtain two matching relationships, as shown by the blue arrow and the red arrow in the figure. Both of these situations may happen, because the scores of the two situations are equal, so the radar pose cannot be estimated correctly. To solve this problem, this paper introduced the RCS feature of the millimeter-wave radar point cloud to further restrict the point cloud matching relationship.

Based on the above two reasons, this paper proposed an improved CSM method:

(1) The improved CSM method no longer matched *Q* with *F*. Instead, it matched *Q* with local submap in global grid map, which is a “scan to map” method.
(2) When constructing the rasterized lookup table, we no longer assigned the same occupation probability to each point in *F* but assigned a different probability value to each point in *F* according to the RCS of the target.

In this section, three improved CSM methods combined with RCS feature were proposed in this Section. In Section 4, abundant experimental data were used to compare and analyze the three methods.

RCS is a parameter to measure the ability of a target to reflect electromagnetic waves. For the same millimeter wave radar, it is related to the material, geometric shape, and observation angle of the target [46]. In other words, the RCS can reflect the characteristics of the target stably when the observation angle is constant. In the practical application of SLAM, because the data rate of the millimeter-wave radar is relatively high, within a short time interval, the observation angle of a static target changes very little. Based on this point, this paper assumed that for the same static target, its observation angle changes little in a short time, so its RCS value is basically unchanged.

Based on the above assumption, the first improved CSM method combined with RCS feature was proposed in this paper. Before performing point cloud matching, the map *M* was constructed first. The map *M* is quantified into a*b grids, and each grid is independent of each other. The value of each grid *M_x_*_,*y*_ in the map is the RCS observed by radar, which is represented by *R*(*M_x_*_,*y*_) in this paper. The update strategy of *R*(*M_x_*_,*y*_) is shown in Formula (9).

(9) $$R(M_{x,y})(t)=\frac{\sum {}_{k=0}^{t}\left(\frac{1}{r_{x,y}(k)}\cdot R(M_{x,y})(k)\right)}{\sum {}_{k=0}^{t}\left(\frac{1}{r_{x,y}(k)}\right)},0\leq k\leq t$$

By calculating the weighted average of all radar observations *R*(*M_x_*_,*y*_)(*k*) up to time *t*, the grid cell value *R*(*M_x_*_,*y*_)(*t*) at time *t* were calculated. The single observation value is the reciprocal weighting of the radial distance *r_x_*_,*y*_(*k*) between the target and the radar. Because of this weight, the near measurement has a stronger influence on the grid map than the remote measurement. This is because considering the angular error of radar measurement, the position error of the target in the Cartesian coordinate system decreases with the decrease of the distance to the target.

The first frame data in the “Multi-scan” after the outliers are filtered is taken out for query scanning *Q*. Match *Q* with the local submap in the grid map. In order to determine the size of local submap, *n* targets (*r_m_^x^*, *r_m_^y^*), 1 *≤ m ≤ n* in *Q* are translated and rotated using the pose *T_t_*_−1_ of radar at the last moment, and the absolute coordinates (*r_m_^x′^*, *r_m_^y′^*), 1 ≤ *m* ≤ *n* of targets are obtained. Then, we obtained the minimum and maximum values of *x* and *y*: *minrx′*, *minrx′*, *minrx′*, and *minrx′*. They initially construct the boundary of local submap. In order to achieve better matching, the threshold *Bodersize* is set to expand the boundary. The final extracted submap is shown in Formula (10).

(10) $$\begin{array}{l} Submap(t)=M_{x,y}(t),minx\leq x\leq maxx,miny\leq y\leq maxy\\ \left\{ \begin{array}{l} minx=\min r^{x\prime }-Bodersize,maxx=\max r^{x\prime }+Bodersize\\ miny=\min r^{y\prime }-Bodersize,maxy=\max r^{y\prime }+Bodersize \end{array}\right. \end{array}$$

At this time, all targets in the submap, namely reference scan *F*, were established rasterized lookup tables. This paper still used Gaussian kernel to blur all targets in submap. The difference is that each target is given a weight, *R*(*mx*,*y*)(*t*), which is the RCS value of the target in the map at this time. Formula (8) was replaced by Formula (11).

(11) $$P(F^{*})=\frac{1}{\sigma \cdot \sqrt{2\pi }}e^{-\frac{{\left(Dist-\mu \right)}^{2}}{2\sigma^{2}}}\cdot R(M_{x,y})(t)$$

**Figure 7:**
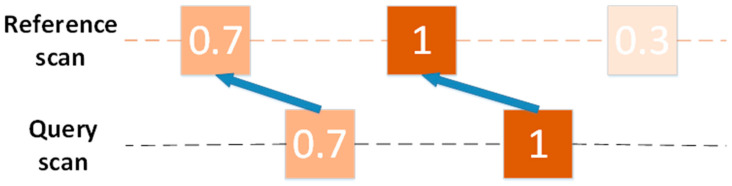
Scanning point cloud of two adjacent frames. The color and number of the square indicate that the target has different RCS. In the end, the only correct matching relationship will be obtained, as shown by the blue arrow.

When estimating the radar pose *T_t_*, the original CSM traverses and queries the scores of all *Q* on the rasterized lookup table and records the maximum value of the total score. However, in this method, it is meaningless to get the maximum total score of RCS, and the maximum total score of RCS does not represent the best match. This paper established another matching strategy. After projecting *Q* to the rasterized lookup table, the RCS of *Q* is subtracted from the RCS of hitting grid, and then the result is summed. Based on the above assumptions, the smaller the sum, the higher the accuracy of matching. The best pose estimation *T_t_* is obtained by obtaining the minimum sum of pose. The matching strategy is shown in Figure 7. The colors and numbers in the box represent the RCS of the target. In order to minimize the sum, the only correct matching relationship can be finally obtained, as shown by the blue arrow. It should be noted that this strategy cannot use the multiresolution search method, so violent matching method ws used to estimate the radar pose *T_t_*.

In Section 2.1, the filtered pose sequence [*O_t_*, *O_t_*_+1_, …, *O_t+N_*] is obtained. Because odometry and IMU only have high accuracy in a short time, they can not be used for a long time. So replace *O_t_* with the estimate *T_t_*. According to the relative relationship between [*O_t_*, *O_t+_*_1_, …, *O_t+N_*], [*T_t_*, *T_t+_*_1_, …, *T_t+N_*] is obtained as the accurate radar pose sequence. When *t =* 0, [*T*_0_, *T*_1_, …, *T_N_*] = [*O*_0_, *O*_1_, …, *O_N_*].

In this paper, the position sequence [*T_t_*, *T_t+_*_1_, …, *T_t+N_*] was used to transform the *N* frames scanning in “Multi-scan,“ and the map was updated with Formula (8). When the next “Multi-scan” arrived, the above steps were repeated. In this paper, the maps and trajectories generated by this method were analyzed with actual data. The results are shown in Section 4.3.

Next, this paper proposed a second improved CSM method combined with RCS. The RCS of the target was replaced by the echo power of the target. According to the radar Equation (12), the echo power of the target is related to the radial distance and RCS, where *P_t_* is the radar transmitting power and *G* is the radar antenna gain. They are constant for the same radar. *r_x_*_,*y*_ represents the radial distance between the target and the radar, *RCS* is the RCS of the target, and *A* represents the echo power of the target.

(12) $$A=\frac{P_{t}G}{4\pi r_{x,y}{}^{2}}\cdot RCS$$

It was also necessary to construct the map *M* before performing point cloud matching. The authors of [46] constructed amplitude grid map, and the map here is the same in essence. The value of each grid *M_x_*_,*y*_ a is the echo power of the target, expressed as *A*(*M_x_*_,*y*_)(*t*). The update strategy for each grid is shown in Formula (13).

(13) $$A(M_{x,y})(t)=\frac{\sum {}_{k=0}^{t}\left(\frac{1}{r_{x,y}(k)}\cdot A(M_{x,y})(k)\right)}{\sum {}_{k=0}^{t}\left(\frac{1}{r_{x,y}(k)}\right)},0\leq k\leq t$$

**Figure 8:**
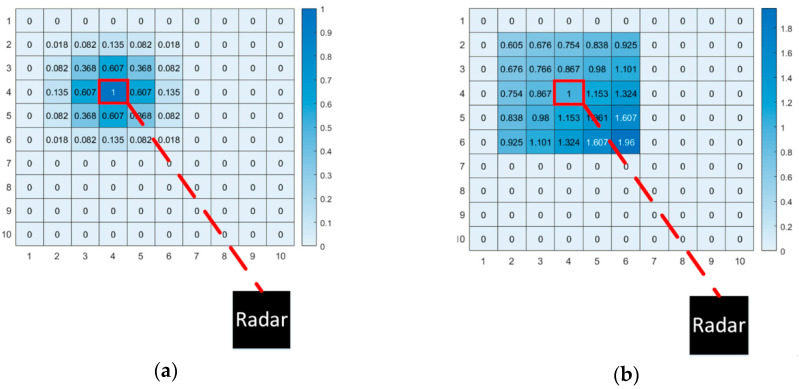
Two rasterized lookup tables. The black squares are radars, and the red squares represent the targets observed by the radar. (**a**) Rasterized lookup table based on occupancy probability. The number in the grid represents the probability of this grid being occupied. (**b**) The lookup table was based on the echo power, and the number in the grid represents the echo power of this grid.

In the same way, query scan *Q* was selected and local submap was selected according to Formula (9). However, the proposed method is different from the original CSM method in establishing a rasterized lookup table for all targets in the submap. The rasterized lookup table constructed by the original CSM method was based on the probability distribution over the Euclidean distance. After the Gaussian kernel blur, the occupancy probability of the target decreased from the inside to the outside, as shown in Figure 8a. However, the echo power *A* of the target no longer conformed to the Gaussian distribution. Based on the radar equation and the above assumptions, when the RCS is basically unchanged, the echo power *A* of the target is related to the radial distance *r_x_*_,*y*_ from the target to the radar. Therefore, a rasterized lookup table was established according to Formula (12), as shown in Figure 8b. It can be seen from the figure that the echo power of the target was fan-shaped, which was inversely proportional to the radial distance *r_x_*_,*y*_ from the target to the radar. This phenomenon is in line with the actual situation.

We still used violent matching method to estimate the radar’s pose *T_t_*. The matching strategy was almost the same as the previous method. *Q* was projected into the rasterization lookup table, and the echo power of *Q* was subtracted from the echo power of hitting the grid, and then the result was summed. The smaller the sum of the differences, the higher the matching degree. The best pose *T_t_* is the position with the smallest sum. Finally, [*T_t_*, *T_t+_*_1_, …, *T_t+N_*] was obtained as the accurate radar pose sequence. We used [*T_t_*, *T_t+_*_1_, …, *T_t+N_*] to convert the *N* frames scan in “Multi-scan,” and updated the map with Formula (13). When the next “Multi-scan” arrived, the above steps were repeated. Using the same actual data as the previous method, the map and trajectory generated by this method were analyzed. The results were shown in Section 4.3.

Finally, this paper proposed the third improved CSM method combined with RCS. This method was no longer based on the above assumption that RCS is basically unchanged, but used RCS features from a different perspective. RCS represents a physical quantity of echo intensity produced by a target irradiated by radar wave. Targets with low RCS are weak reflectors, while targets with high RCS are strong reflectors. The lower the RCS of a reflector, the smaller the echo intensity it produces, and the less likely it will be detected by radar.

First, construct the map *M*. We referred to the occupied grid map (OGM) constructed by the authors of [46] for millimeter-wave radar. In this paper, binary Bayesian filtering was used to update each grid of the map, such as Formula (14), where *L*(*M_x_*_,*y*_(*t* − 1)) is the probability of the last time on the grid, *L*(*M_x_*_,*y*_(0)) is the probability of the initial time of the grid (*L*(*M_x_*_,*y*_(0)) = 0), and the last term of the formula is called the anti-sensor model.

(14) $$L(M_{x,y}(t))=L(M_{x,y}(t-1))-L(M_{x,y}(0))+\log\frac{P(M_{x,y}(t)|z_{t},x_{t})}{1-P(M_{x,y}(t)|z_{t},x_{t})}$$

Different from paper [46], this paper took the RCS feature as the weight of occupancy probability of each grid and replaced Formula (14) with Formula (15). Referring to [43], the surface normal of a point was used as an additional feature. The anti-sensor model in Formula (13) assigns the same occupancy probability to each point in the point cloud, while the anti-sensor model in Formula (14) weakens the occupancy probability of low RCS targets. In other words, this formula enhances the occupancy probability of strong reflectors and weakens the occupancy probability of weak reflectors. In Formula (14), *RCS_x_*_,*y*_(*t*) represents the RCS value of the grid observed by radar at *t* time.

(15) $$L(M_{x,y}(t))=L(M_{x,y}(t-1))-L(M_{x,y}(0))+\log\frac{p(M_{x,y}(t)|z_{t},x_{t})}{1-p(M_{x,y}(t)|z_{t},x_{t})}\cdot RCS_{x,y}(t)$$

Use Formula (15) to update the map. In this paper, the map *M* was called RCS occupied raster map (R-OGM). As the two methods mentioned above, query scan *Q* and local submap were selected, and then the rasterization look-up table was established. It should be noted that although the RCS feature of the target was used as the weight, the map itself was constructed based on the occupation probability. Therefore, the Gaussian kernel was still used to blur all targets in the submap. The difference is that each target is given a weight, *L*(*M_x_*_,*y*_(*t*)), which is the value of the target in R-OGM at this time. Therefore, Formula (16) was used instead of Formula (7).

(16) $$P(F^{*})=\frac{1}{\sigma \cdot \sqrt{2\pi }}e^{-\frac{{\left(Dist-\mu \right)}^{2}}{2\sigma^{2}}}\cdot L(M_{x,y}(t))$$

**Figure 9:**
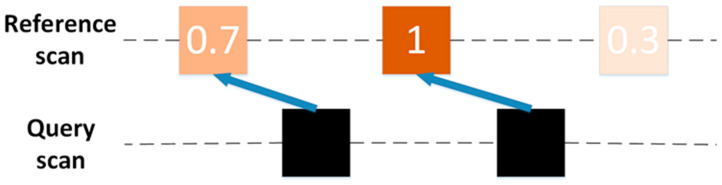
Scanning point cloud of two adjacent frames. In the reference scan, the color and number of the square represent the target with different occupation probabilities. In the end, the only correct matching relationship is obtained, as shown by the blue arrow.

Next, the multiresolution method was used to estimate the radar pose *T_t_*. The score of query scan *Q* in the rasterized lookup table was calculated and summed to obtain the total score. The pose with the highest total score is the best pose estimation *T_t_*. The matching strategy is shown in Figure 9. In the reference scan, the number and color of the block indicate the occupation probability of the target. Because of the RCS weight, the occupancy probability of different targets is different. Thus, the unique maximum total score can be obtained, which corresponds to the correct matching relationship, as shown by the blue arrow in the figure. Finally, the accurate radar pose sequence [*T_t_*, *T_t+_*_1_, …, *T_t+N_*] can be obtained. Use [*T_t_*, *T_t+_*_1_, …, *T_t+N_*] to convert *N* frames scan in “Multi-scan”, and update the map with formula (12). When the next “Multi-scan” arrives, repeat the above steps. This paper used the same actual data as the above two methods to analyze the map and trajectory generated by this method. The results are shown in Section 4.3.

## 4. Results and Discussion

### 4.1. Experimental Platform and Scene

This paper used a mobile platform that integrates Odometry, IMU and a 77GHz millimeter-wave radar for data collection. The detection distance of the radar was 0–250 m, the detection angle was ±60°, the distance measurement accuracy was 0.05–0.4 m, the angle measurement precision was ±0.1°–±5°, the distance measurement resolution was 0.2–1.79 m, and the angle measurement resolution was 1.6°–12.3°. The data refresh rate of the radar was 13 Hz. All data was processed on a laptop equipped with MATLAB R2019b, the CPU was 1.99 GHz Intel Core i7-8565U, and the RAM was 8 GB.

The two main radar datasets are the Multimodal range dataset (Mulran) [47] and Oxford radar robotcar dataset [48]. They greatly facilitate the study and research of the majority of scientific researchers. They have both the ground truth and diverse scenarios. However, compared with them, the radar data refresh rate used in this paper is higher (the radar in this paper is 13 Hz, and the radars in both datasets are 4 Hz), which is necessary under some special conditions.

**Figure 10:**
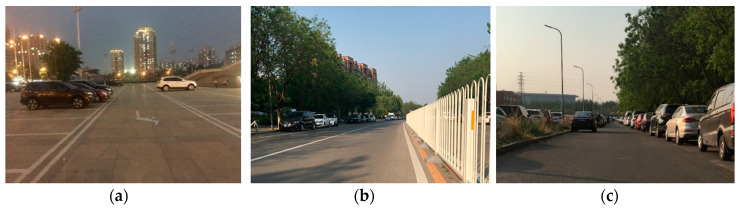
Three experimental scenes obtained by optical cameras (**a**) scene 1; (**b**) scene 2; (**c**) scene 3.

This paper collected three groups of experimental data, and the experimental scene is shown in Figure 10. Figure 10a shows scene 1, which was a ground parking lot. There were no moving targets in the scene, and there were only a few parked cars. The control radar circled the three cars in the scene. The scene was relatively empty, and it was a degraded environment, which was not conducive to point cloud matching. This scene was to test the robustness of radar SLAM in degraded environments. Figure 10b shows scene 2, which was a street in the suburbs of a city. The stationary objects in the scene included trees, guardrails, vehicles, and passersby. A few moving targets included pedestrians, bicycles, and moving cars. This scenario was challenging for the radar. The scene contained a lot of weak reflectors (trees and pedestrians) and strong reflectors (guardrails). Weak scattering objects were not easy to be detected by radar, but guardrails were prone to multipath effects because they are too dense. Figure 10c is scene 3, which was a relatively ideal scene. On both sides of the road, a large number of stationary vehicles provided stable and rich geometric information for the environment. It provided basic verification for the entire SLAM. However, because the strong reflectors (cars) were too dense, it was also prone to multipath effects.

### 4.2. Data Preprocessing Results

In this section, the radar data preprocessing method proposed in this paper was analyzed and verified. The data preprocessing link consisted of three steps: ROI to filter out low-precision points, Odometry and IMU combination “Multi-scan”, and DBSCAN to filter out outliers. In the summary of Section 2.1, this article compared the difference between single-frame radar data and “Multi-scan”. Through Figure 3, it could be seen that the amount of point cloud data significantly increased, and the point cloud density also significantly increased. So, we focused on analyzing the effect of screening out low-precision points and screening out outliers.

In this paper, when using DBSCAN to filter out outliers, a vector *X_m_* = *[r_m_^x^*, *r_m_^y^*, *rcs_m_*] of three-dimensional features of radar data was constructed. The Mahalanobis distance was used to measure the similarity of the vectors and the adaptive determination of the parameters was completed. Generally, when using DBSCAN to process radar data, only the vector of two-dimensional features of the radar data was usually constructed: *X_m_* = *[r_m_^x^*, *r_m_^y^*], and the Euclidean distance was used for the similarity measurement. Therefore, in this section, we compared the recognition results of outliers with and without RCS features. Both methods used the parameter adaptive determination method that was proposed in this paper to determine the parameters.

**Figure 11:**
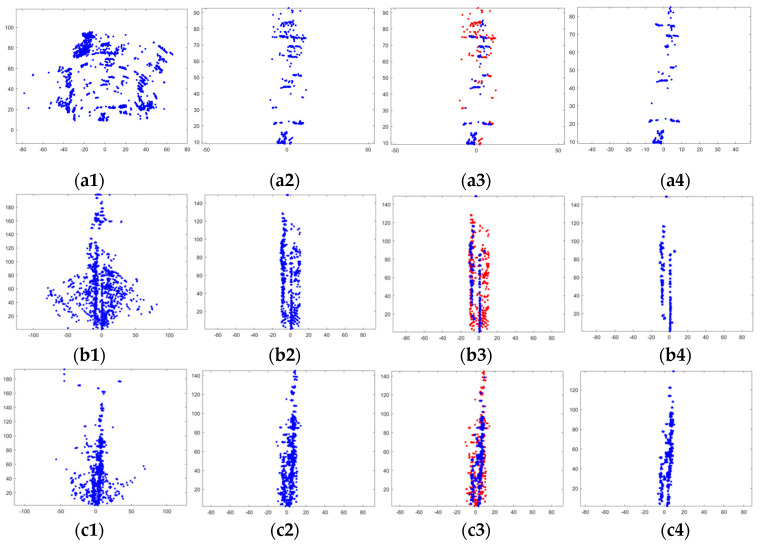
The three groups of pictures (**a**–**c**) correspond to three scenes. (**a1**–**c1**) A point cloud map combining 20 frames of original data; (**a2**–**c2**) A point cloud map after filtering out low-precision points; (**a3**–**c3**) An effect map of DBSCAN identifying outliers, in which the red points are outliers; (**a4**–**c4**) Point cloud after filtering out outliers. The vertical direction is the y-axis, and the horizontal direction is the x-axis.

First, this paper verified the effect of each step in the preprocessing link, as shown in Figure 11. The figure includes the processing results of three groups of data, a, b, and c, corresponding to three scenes, respectively. a1–c1 are the point cloud images of the original data. a2–c2 is the point cloud after using ROI to filter out low-precision points. a3–c3 are the results of DBSCAN recognition of outliers using three-dimensional features. a4–c4 is the point cloud after filtering out outliers, and the final output of the original data after preprocessing. It should be noted that the actual sequence of the preprocessing step was to filter out low-precision points first, and then construct the “Multi-scan”. This can reduce a large part of the calculation. However, in order to fully reflect the characteristics of the original data, a “Multi-scan” was constructed and then ROI was used to filter out low-precision points.

By observing a1–c1, it can be found that the original point cloud of the radar presented a fan shape. This is because the radar always emits fan-shaped electromagnetic waves to the front when it is working. So, the radar’s detection range was ultimately fan shaped. Observing b1 and c1, the point cloud distribution at the edge of the fan was very sparse relative to the inside of the fan. When we observe a1, although the point cloud at the edge of the fan was no longer sparse, it can be seen that these point clouds were distributed in an arc shape. The reason for this phenomenon was that the radar’s range and angle measurement precision was low at the edge of the fan. These low-precision points cannot describe the geometric characteristics of the environment well. They only increase the error of point cloud matching and need to be filtered out. After using ROI to filter the original data, observing a2–c2, it can be seen that most of the low-precision points were filtered out. It verified the effectiveness of using ROI to filter out low-precision points.

Using DBSCAN with RCS feature to identify the outliers in a2–c2, the result was as a3–c3, the red points in the figure were the identified outliers, and the blue points were the reserved point cloud. After the outliers were removed, the result was shown in Figure 11(a4–c4). We can observe the two straight lines in b4 and c4, which corresponded to the guardrail and the parked car in Figure 10b. The two straight lines in c4 corresponded to the cars parked on both sides of the road in Figure 10c. Comparing b4, c4 and b2, c2, it can be seen that the geometric outline of the environment became clearer. So, we know that most of the outliers were indeed effectively screened out. Comparing a4 and a2, the change was not obvious, because scene 1 itself was empty. The environment itself provided very little geometric information, so the point cloud detected by radar was also reduced. The experimental results in this part verified the effectiveness of using DBSCAN to screen out outliers.

**Figure 12:**
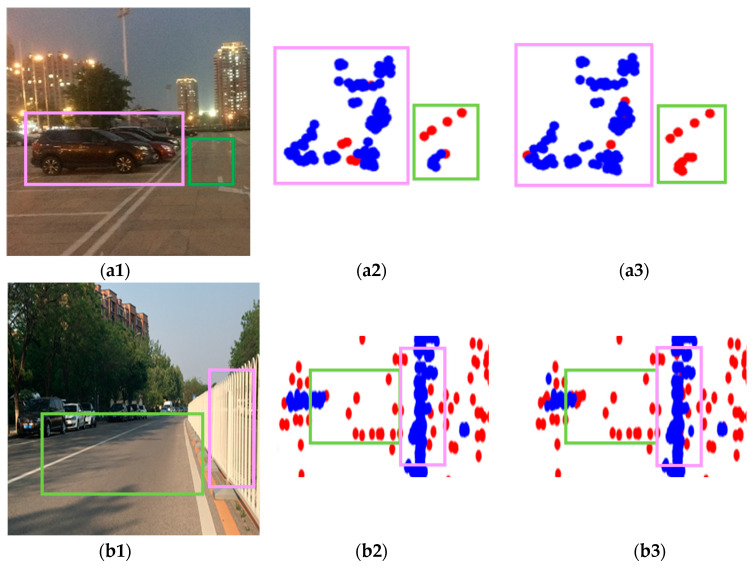

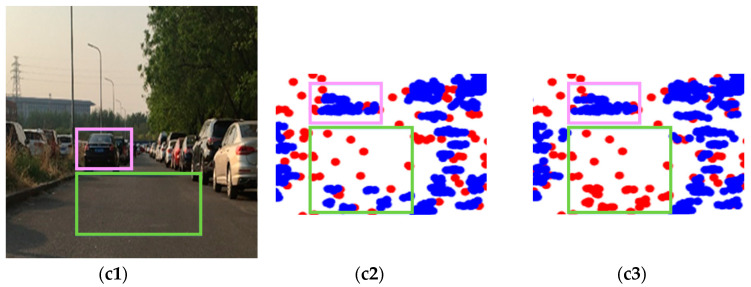
The three sets of pictures. (**a**–**c**) correspond to three scenes. (**a1**–**c1**) Partial image of the real scene; (**a2**–**c2**) Outlier recognition result when RCS feature is not used, red is outlier; (**a3**–**c3**) Outlier recognition result when RCS feature is used, red is outlier.

Then, we compared the recognition results of outliers using RCS features and DBSCAN without RCS features, as shown in Figure 12. The figure included the processing results of three groups of data, a, b, and c, corresponding to three scenes, respectively. a1–c1 were partial images of the real scene. a2–c2 were the partial results of outlier recognition without RCS features. a3–c3 were the partial results of outlier recognition using RCS features. The *e* and *MinPts* parameters of the two methods were determined using the method proposed in this paper. In the three pictures of each scene, the pink frame and the green frame corresponded one to one. For the pink box, we can observe that there were three cars in a1, a row of guardrails in b1, and one car in c1. Both a2–c2 and a3–c3 could retain the main point cloud of these targets, and there was almost no difference between the results of using RCS features and not using RCS features. Regarding the green frame, the green frames in the three scenes were all open spaces, and the point clouds in this area were all outliers. Observing b2 and b3, the point clouds in the green box were all identified as outliers, which was consistent with the actual situation. However, in a2 and c2, some of the point clouds in the green boxes were not recognized as outliers, while the point clouds in the green boxes in a3 and c3 were all recognized as outliers. The reason for the poor recognition results without using RCS features was that the location distribution of these outliers was uneven. Sometimes, the point cloud could not be accurately divided by relying only on the Euclidean distance without using the RCS feature of the target. After adding the target’s RCS feature, we found that this situation had been improved. The reason was that this method used more information of the target to divide the point cloud and eliminated the interference of uneven distribution of outliers. In summary, the recognition results of outliers using RCS and Cartesian three-dimensional features were better than the results of outlier recognition without RCS features.

### 4.3. Trajectory and Map

In this section, first, the three improved CSM methods combining RCS features proposed in Section 3.2 were analyzed and verified. In order to simply compare the pros and cons of the three methods, clustering was not performed here, and the aid of IMU and Odometry was not used. This paper compared the map and the trajectory respectively. When comparing maps, the true value of the radar track was required. Since the experiment had no true value, this paper used a group of best estimated trajectories instead of the true trajectories. When comparing trajectories, the entire trajectory was not compared. Only the trajectory of the first 180 frames was compared, because this part of the trajectory can already explain the pros and cons of the algorithm.

**Figure 13:**
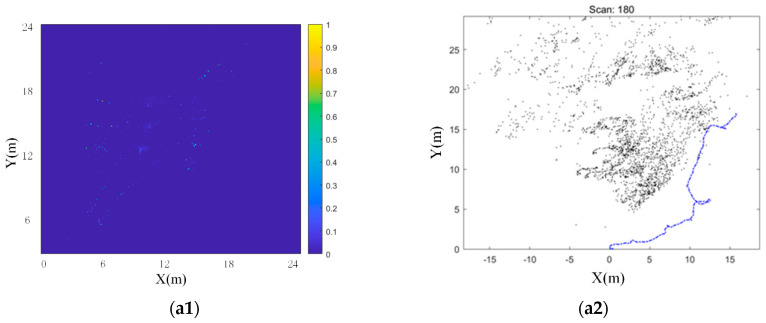

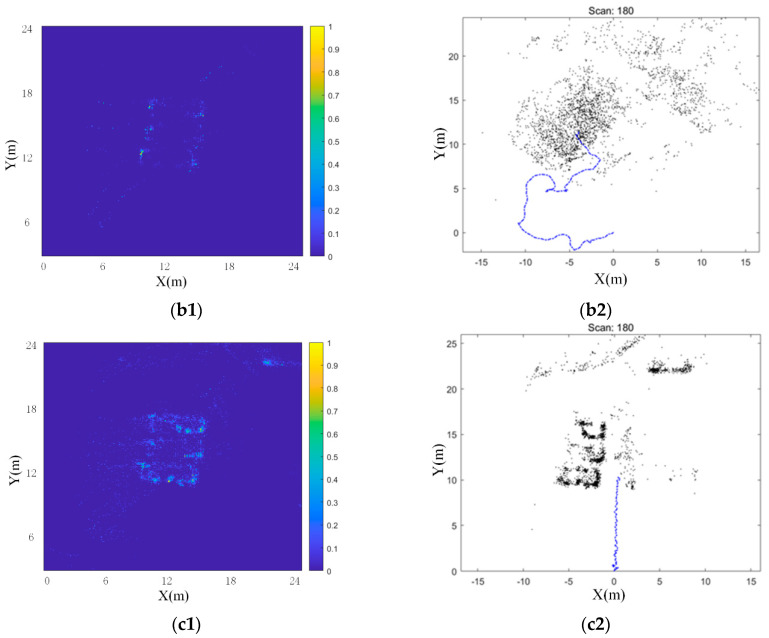
The maps and trajectories generated by the three improved CSM methods combining RCS features. (**a1**–**c1**) Maps generated by three methods. The three maps correspond to different physical quantities. (**a1**) corresponds to the RCS of the target, (**b1**) corresponds to the echo power of the target, and (**c1**) corresponds to the existence probability of the target. The dark blue corresponds to the minimum value of the physical quantity, and the light yellow corresponds to the maximum value of the physical quantity. (**a2**–**c2**) Trajectories generated by the three methods when only 180 frames of data are used. The blue line is the radar trajectory, and the black point is the radar point cloud.

For scenario one, the radar circled three cars. The map and trajectory generated by method one are shown in Figure 13(a1 and a2). The map and trajectory generated by method three are shown in Figure 13(c1 and c2). The maps generated by the three methods corresponded to different physical quantities. A1 corresponded to the RCS of the target, b1 corresponded to the echo power of the target, and c1 corresponded to the existence probability of the target. Dark blue corresponded to the minimum physical quantity, and light yellow corresponded to the maximum physical quantity. In a2–c2, the blue line was the radar track, and the black point was the radar point cloud.

When comparing maps, we observed a1, b1, and c1. It was hard to see the existence of three cars in a1. The number of target points seen by the naked eye in a1 was very small, because method one weakened the expression of low RCS targets in the map. At the same time, because the method weighted the reciprocal of the distance to each target point, it also weakened the expression of distant target points. A2 performed better than a1, as a2 could better describe the existence of three cars in the map, especially more sensitive to the outline of the car. The reason is that the edges of the car could generate more echo power. The number of target points seen by the naked eye in a2 was also very small, because the second method weakened the expression of low echo power points. At the same time, as the second method also weighted the reciprocal of distance for each target point, it also weakened the expression of distant target points. Observing a3, we can clearly see the existence of three cars. The performance of a3 was significantly better than a1 and a2. In this paper, the map generated by method 3 was called R-OGM.

When comparing trajectories, we observed a2, b2, and c2. Obviously, method three could correctly estimate the trajectory of the radar, but the trajectory generated by method one and method two failed. We reviewed each step of method one and method two, and believed that the reason for positioning failure appeared in the step of constructing the rasterized lookup table. The physical quantity of the map constructed by method one corresponded to the RCS of the target. Method one used Gaussian kernel to blur the RCS of the target, which was unreasonable. Because the RCS of the target had nothing to do with distance, and the Gaussian kernel was only related to distance. In addition, the assumption in Section 3.2 that the RCS of the target was basically unchanged only in a small observation angle range, not 360°, was incorrect. When the radar made a full circle around the car, it actually made a 360° observation of the target. The RCS fluctuation of the target was violent and irregular, as described by the authors of [49,50]. Therefore, it was wrong to use Gaussian kernel in method one when blurring the RCS of the target. The physical quantity of the map constructed by method two corresponded to the echo power of the target. In the same way, although the second method used radar equations to obscure the target’s echo power in the radial distance, it did not consider the change of the echo power in a wide range of angles, so method two was also imperfect. Although method 3 used the RCS feature of the target as the weight, the map itself was constructed based on the occupation probability. Therefore, it was reasonable to use a Gaussian kernel to construct a rasterized lookup table.

The above content only discussed the experimental results of scene one, but the results of scene two and scene three were similar. The analysis of the above data had been able to preliminarily prove the effectiveness of the improved CSM (referred to as Method three below). Then, this paper used all three groups of experimental data to conduct a more detailed analysis and verification of the improved CSM and the millimeter-wave radar SLAM proposed.

**Figure 14:**
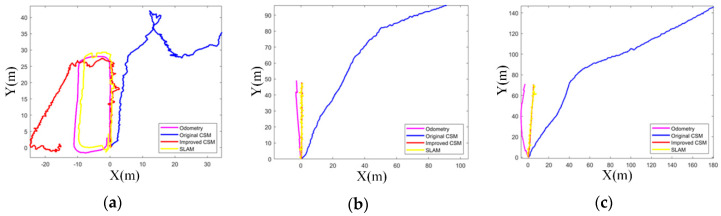
The three figures (**a**–**c**) correspond to the trajectories of the three scenes.

First, we compared the trajectories, as shown in Figure 14. The three pictures a, b, and c corresponded to three experimental scenes. In each scene, the trajectory generated by Odometry, the trajectory generated by the original CSM, the trajectory generated by the improved CSM, and the trajectory generated by the entire SLAM were compared. The trajectory of Odometry was generated from the original data of Odometry. When generating the trajectories of the original CSM and the improved CSM, in order to simply compared the quality of the point cloud matching method, Odometry information was not used. Therefore, no “Multi-scan” was constructed, and DBSCAN was not used to filter out outliers. The trajectory of SLAM was generated by the SLAM proposed in this paper. It underwent a complete data preprocessing link and used an improved CSM as a point cloud matching method.

**Figure 15:**
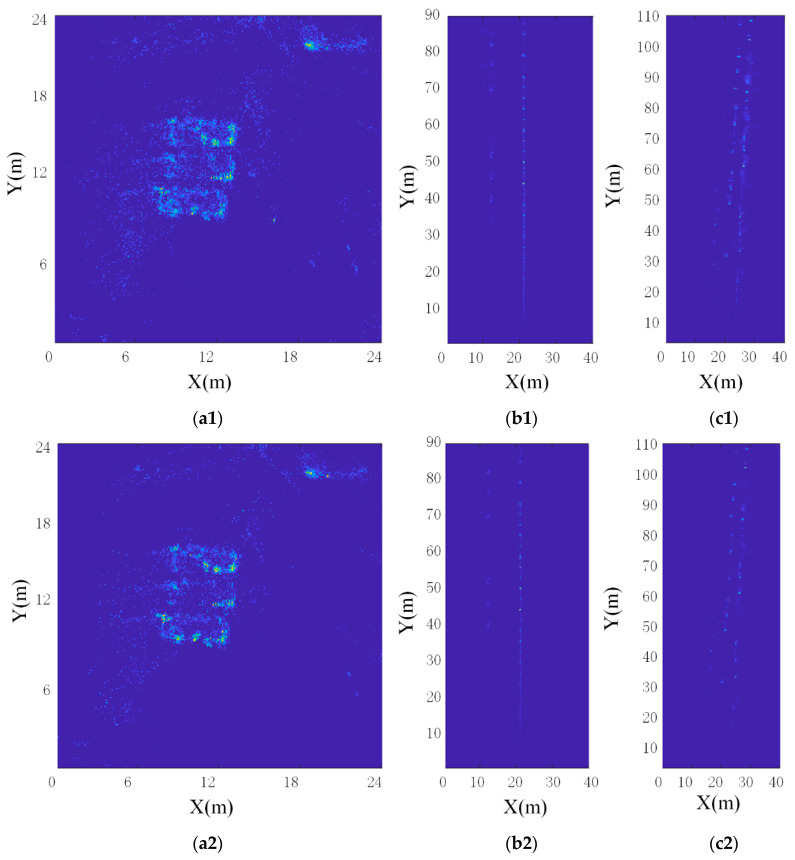
The three sets of pictures (**a**–**c**) correspond to the maps of the three scenes. Among them, (**a1**–**c1**) are OGM and (**a2**–**c2**) are R-OGM.

Due to the lack of the true value of the trajectory, this paper evaluated the quality of the trajectory by comparing the maps. In scene one, the best trajectory was generated by SLAM, and the map constructed with it was shown in Figure 15(a1). The accuracy order of each group of trajectories in scenario one was: SLAM > Odometry > improved CSM > original CSM. In scenario two, the best trajectory was generated by the improved CSM, and the map constructed with it was shown in Figure 15(b1). The accuracy order of each group of trajectories in scene 2 was: Improved CSM > SLAM > Odometry > original CSM. In scene three, the best trajectory was generated by the improved CSM, and the map constructed with it was shown in Figure 15(c1). The accuracy order of each group of trajectories in scene three was: Improved CSM > SLAM > Odometry > original CSM.

It could be seen from Figure 14 that the original CSM trajectory had the worst performance in the three scenes due to the characteristics of the radar data. The precision volume of millimeter-wave radar data was much lower than that of lidar, so the original CSM was not suitable for millimeter-wave radar data. The trajectory of Odometry did not perform well in the three scenes. This was due to hardware precision and road environment. As the movement time increased, the cumulative error of Odometry would gradually increase.

In scene one, the trajectory of SLAM was the best, and the improved CSM ranked second. The reason is that scene one was a degraded environment, and the radar could not scan enough point clouds, which affected the result of point cloud matching. After the radar had driven more faster than three cars, the environment in front did not provide a point cloud for the radar, so the improved CSM method failed. However, SLAM had better robustness because it integrated Odometry information. When the radar scanned three cars again, the improved CSM was a “Scan to Map” matching method, the radar could gradually correct to the correct pose. This was similar to the working principle of closed loop detection. In scenario two and scenario three, the trajectory of the improved CSM was better than that of SLAM. It proved the accuracy of the improved CSM method proposed in this paper. The reason for the poor performance of SLAM lied in the fusion of Odometry information. When constructing “Multi-scan”, this paper directly used the pose data output by Odometry. It brought errors to SLAM due to ground slippage and hardware accuracy. Data fusion errors could be reduced through better fusion methods or graph optimization methods.

In order to accurately analyze the map, the best estimated trajectory was used to construct the map. Scene one used the trajectory of SLAM. Scene two and scene three both used the trajectory generated by the improved CSM. Because the R-OGM established in this paper was a modification of OGM, so the paper also compared the two maps. The map of the three scenes was shown in Figure 15. The three groups of pictures a, b, and c corresponded to three experimental scenes. Among them, a1–c1 were OGM and a2–c2 were R-OGM. Observing OGM and R-OGM, we could see that both types of maps could well reflect the existence of the target in the map. The two straight-line traces in b1 and b2 corresponded to the guardrail and parked cars in Figure 10b. The brighter dots represented the guardrail, because the echo intensity of the guardrail was stronger than that of the car. The traces in c1 and c2 corresponded to the cars parked on both sides of the road in Figure 10c. Compared with OGM, the advantage of R-OGM was that it highlighted the geometric contour of the target. The reason was that R-OGM enhanced the expression of strong reflectors in the map while suppressing the expression of weak reflectors in the map. This was beneficial for landmark extraction and matching with offline maps. However, for path planning, this was unfavorable, because even a small target with a probability cannot be ignored.

### 4.4. Data Volume

**Figure 16:**
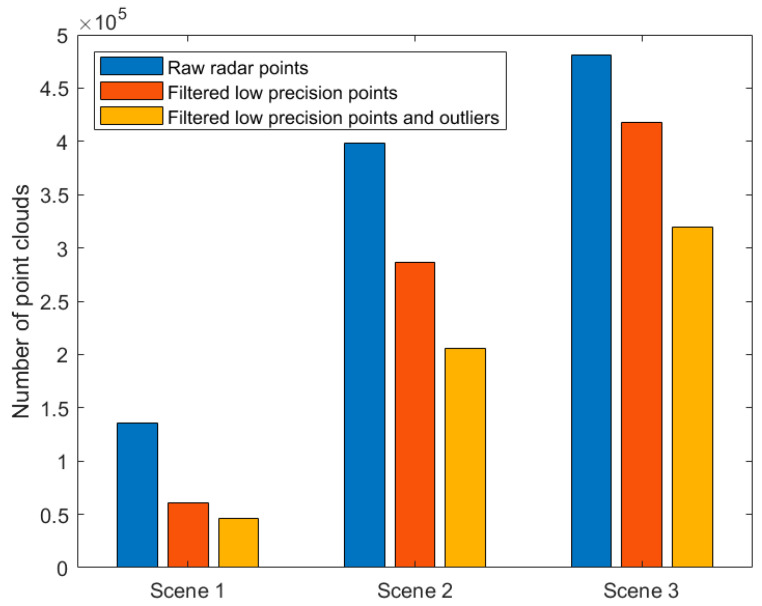
Data volume of the system. The number of point clouds in the original data of the three scenes is compared, the number of point clouds after filtering out low-precision points, and the number of point clouds after filtering out low-precision points and outliers.

In this section, we analyzed the amount of data carried by SLAM. Because the millimeter-wave radar SLAM proposed in this paper eliminated low-precision points and outliers in the data preprocessing step, it greatly reduced the amount of calculation and storage of the computer. In the three experiments, this paper separately counted the number of original point clouds, the number of point clouds after filtering out low-precision points, and the number of point clouds after filtering out low-precision points and outliers, as shown in Figure 16.

Figure 16 visually showed that after the original data was preprocessed, the number of point clouds had dropped significantly. The contribution of each step was further analyzed, as Table 2 shows.

It can be seen from Table 2 that the number of original point clouds of the three scenes was consistent with the actual situation of the scene. The number of original point clouds was related to the environment and the time of radar acquisition. Scene one itself was relatively empty, and the radar acquisition time was the shortest, so the number of original point clouds in scene one was the least. Scene three contained a large number of targets, and the radar acquisition time was the longest, so scene three had the largest number of original point clouds. This paper calculated the ratio of the number of low-precision points to the number of original point clouds. The ratio of scene one was the highest, and the ratio of scene three was the lowest. The number of low-precision points mainly depended on the location of the target in the scene. This paper calculated the proportion of the number of outliers to the number of point clouds after filtering out low-precision points. It could be seen from Table 2 that the proportion of outliers was between 20–30%. The proportions of scene one and scene three were basically equal. The proportion of scene two was slightly higher. Finally, the total number of points to be filtered out was calculated, and the proportion relative to the number of original point clouds was calculated. It could be seen that the most low-quality point clouds were screened out scene one, and the screening rate reached 65.91%. The least low-quality point clouds were screened out scenario three, and the screening rate reached 33.57%. In summary, the millimeter-wave radar SLAM proposed in this paper could effectively reduce the computer’s calculation and storage load.

## 5. Conclusions

In this paper, a millimeter-wave radar SLAM assisted based on RCS and IMU assistance was proposed, which includes radar data preprocessing and tightly coupled localization and mapping. In each parts of the proposed method, the assistance of RCS target was used. In data preprocessing, this paper selected ROI to filter out low-precision points and used DBSCAN to filter outlier points. When DBSCAN filtered outliers, this paper used the three-dimensional features of radar data: The Cartesian position of the target and RCS. This paper also used the Mahalanobis distance to measure the similarity of the radar data, which realized the adaptive determination method of DBSCAN parameters. In localization and mapping, this paper proposed an improved CSM method. This was a “Scan to Map” point cloud matching method, which achieved the tight coupling of localization and mapping. When constructing the map, this paper used the RCS target, and the constructed map was called R-OGM. In this paper, three improved CSM methods combining RCS target were proposed, and the effectiveness of the third method was verified.

In addition, three groups of actual data were used to verify the proposed method. These three groups of data came from different scenes, which included an empty degraded environment, challenging environment, and the ideal environment for radar. This paper analyzed the results of filtering out low-precision points and outliers. When analyzing the results of filtering outliers, this paper compared the DBSCAN results without RCS feature, and verified that the use of RCS features can identify and screen outliers more accurately. When analyzing the positioning results, this paper compared the trajectory results of Odometry, original CSM, improved CSM and SLAM, verified that the improved CSM method could effectively improve the accuracy of localization, and at the same, time verified the robustness of SLAM proposed in this paper when facing degraded environments. When analyzing the map, the OGM and R-OGM proposed in this paper were compared to verify that the R-OGM can highlight the geometric outline of the target. Finally, this paper analyzed the amount of data generated by the system. Compared with the data volume of the original point cloud, the SLAM proposed in this paper could effectively reduce the computing and storage load of the computer.

In future work, we will consider a better Odometry fusion method. In addition, the millimeter-wave radar SLAM proposed in this paper does not include back-end optimization, so the future work will consider using graph optimization to further modify the pose. These work will further improve the accuracy of localization and mapping.

## Figures and Tables

**Table 1:** Measurement range and precision of the millimeter-wave radar.

Radar Detection Parameters	Parameter Range
Distance range	0.20...150 m@0…±9° far range, 0.20...70 m@0…±45° near range, 0.20…20 m@±60° near range
Precision distance measuring	±0.40 m far range, ±0.10 m (±0.05 m@standstill) near range
Precision azimuth angle	±0.1° far range, ±0.3°@0°/±1°@±45°/±5°@±60°near range

**Table 2:** Statistics of the screening rate of low-precision points and outliers.

Scenes	Raw Radar Points (RPP)	Low-Precision Points (LPP)	Percentage (LPP/RPP)	Outliers (O)	Percentage (O/(RPP − LPP))	Sum (LPP + O)	Percentage (Sum/RPP)
Scene 1	135,318	75,001	55.43%	14,182	23.51%	89,183	65.91%
Scene 2	397,999	111,165	27.93%	80,669	28.12%	191,834	48.20%
Scene 3	480,844	63,370	13.17%	98,033	23.48%	161,403	33.57%
